# Sanitizing Hatching Eggs with Essential Oils: Avian and Microbiological Safety

**DOI:** 10.3390/microorganisms11081890

**Published:** 2023-07-26

**Authors:** Gabriel da Silva Oliveira, Concepta McManus, Maria Viviane de Araújo, Davi Emanuel Ribeiro de Sousa, Isabel Luana de Macêdo, Marcio Botelho de Castro, Vinícius Machado dos Santos

**Affiliations:** 1 Faculty of Agronomy and Veterinary Medicine, University of Brasilia, Brasilia 70910-900, Brazil; gabriels.unb@gmail.com (G.d.S.O.);; 2 Laboratory of Poultry Science, Federal Institute of Brasilia—Campus Planaltina, Brasilia 73380-900, Brazil

**Keywords:** economic gains, egg disinfection, embryological safety, egg microbiology, poultry health, poultry production

## Abstract

Increased meat and egg production leads to concomitant changes in poultry practices, including the indiscriminate use of formaldehyde to sanitize hatching eggs. Although this sanitizer aids in the increase in poultry production, its toxic potential for man and for avian embryos represents an obstacle to its long-term use. This review assesses whether essential oils fit into the context of hatching egg contamination, reviewing their antimicrobial efficiency, toxicity to poultry embryos and chicks, and their sanitizing effects on poultry production parameters. Studies have indicated that, because they are safer, most of the essential oils studied can be a potential substitute for formaldehyde for minimizing microbial exposure of hatching eggs and embryos. However, complementary studies on the microbiological profile of embryos and chicks hatched from eggs sanitized with essential oils need to be carried out and the economic feasibility of the candidate products should also be considered.

## 1. Introduction

The large number of healthy embryos that hatch supports the hypothesis that eggs have good microbiological quality. Ensuring embryo safety in the face of microbiological challenges is not easy. The embryo’s immature status makes it insecure and defenseless against infection [1]. In this case, the eggshell can have a negative effect because it contains pathogenic microorganisms [2] and has communication routes with the embryo, favoring contact between them. Therefore, the quest for healthier poultry is increasing the need to incubate eggs with minimal microbial loads in poultry hatcheries during all incubation cycles. In this case, sanitizing hatching eggs with liquid or gas is the gold standard method of achieving this goal [3]. The sanitization of hatching eggs is nothing more than an antimicrobial resource intermediated by a simple or complex system (e.g., fumigation, spraying, or immersion) that applies a sanitizing solution to the eggshells to solve poultry losses caused by microorganisms. This step must occur within half an hour after laying or immediately collection [4,5,6].

**Figure 1:**
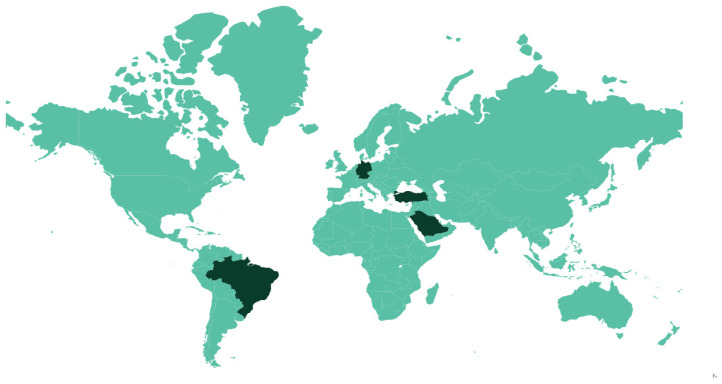
Countries (highlighted in dark green) that most published papers studying essential oils as sanitizers for hatching eggs between 1970 and 2022. Search format performed in SCOPUS: (TITLE-ABS-KEY (sanitizers AND for AND hatching AND eggs) OR TITLE-ABS-KEY (disinfectants AND for AND hatching AND eggs) OR TITLE-ABS-KEY (sanitization AND of AND hatching AND eggs) OR TITLE-ABS-KEY (disinfection AND of AND hatching AND eggs) OR TITLE-ABS-KEY (decontamination AND of AND hatching AND eggs)). Of the 238 papers found, 89 evaluated sanitizers for hatching eggs and of these 13 involved essential oils. The information was collected on 15 February 2023.

**Figure 2:**
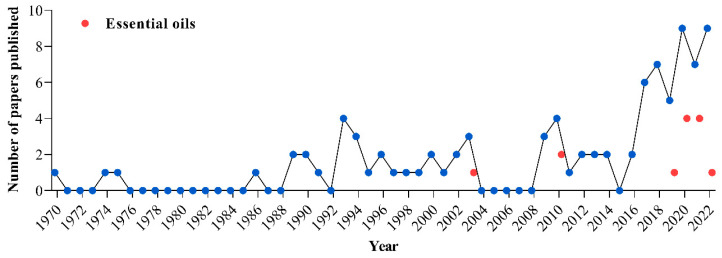
Number of published studies on the use of essential oils as sanitizers for hatching eggs (red ball) compared to the number of published studies that evaluated other sanitizers for hatching eggs (blue ball) in the period 1970 to 2022. Search format performed in SCOPUS: (TITLE-ABS-KEY (sanitizers AND for AND hatching AND eggs) OR TITLE-ABS-KEY (disinfectants AND for AND hatching AND eggs) OR TITLE-ABS-KEY (sanitization AND of AND hatching AND eggs) OR TITLE-ABS-KEY (disinfection AND of AND hatching AND eggs) OR TITLE-ABS-KEY (decontamination AND of AND hatching AND eggs)). Of the 238 papers found, 89 evaluated sanitizers for hatching eggs and of these 13 involved essential oils. The information was collected on 15 February 2023.

In line with the current trend towards ecologically friendly products with minimal impact on animals, the poultry industry needs to gradually adopt sanitizers that respect safety criteria for the protection of avian life. In a previously published review, Oliveira et al. [3] showed that there are various sanitizers for hatching eggs that are available to the poultry industry which are divided into two large groups (synthetic and natural). Among the natural options recommended to the industry, the authors show that essential oils derived from volatile liquids from aromatic plants are antimicrobial and safe to use. The use of essential oils as sanitizers for hatching eggs was reviewed by Oliveira et al. [5]. They reported that essential oils compete with synthetic materials for reasons that are of interest to the poultry industry, including embryo and human safety, the ability to control microorganisms in the eggshell, and increased production rates. These effects can be seen at low concentrations, which may overcome the disadvantages of essential oils where they are more expensive than synthetic compounds that require higher concentrations for effective action. Thus, validating the potential and advantageous characteristics of essential oils in the management of hatching eggs can open an important path for their inclusion in the official list of sanitizers used in poultry routines around the world. This is still a limited field of research. A search carried out in the SCOPUS database showed that, between 1970 and 2022, 89 papers were published evaluating sanitizers for hatching eggs, including research, reviews, conference papers, and book chapters written in English, French, Portuguese, and Russian. Of the 89 papers, only 13 were papers that studied essential oils for hatching eggs, and these came from Brazil (5), Turkey (3), Saudi Arabia (3) and Germany (2) (Figure 1), with 76.92% (10) published between 2019 and 2022 (Figure 2).

The current preventive measure based on formaldehyde to reduce the microbial load of hatching eggs commercially is not friendly to any living organism and its replacement can achieve sustainability in egg sanitization. In this sense, it is important to assess whether essential oils can really fit into the context of decontamination of hatching eggs. Therefore, this study reviewed the antimicrobial efficiency of essential oils and their toxicity in poultry embryos and chicks, as well as their sanitizing effects on poultry production parameters. To better support our discussions and cover gaps in the application of essential oils as sanitizers for hatching eggs, any study involving the direct or indirect relationship between hatching eggs and essential oils, as well as table eggs and essential oils, was reviewed.

## 2. Paper Search Method

In addition to the search performed via Scopus (mentioned earlier in the introduction), Google Scholar was used to search for the papers reviewed in this study. Several search terms were used to find the research and review papers, book chapters and books that fit the proposed content for each topic, including contamination of hatching eggs, chicken embryo infection, sanitizers for hatching eggs, essential oils, antimicrobial activity of essential oil, essential oil for hatching eggs, eggs and essential oils, essential oil and chicken embryos and formaldehyde for hatching eggs. These terms were also searched in Portuguese when necessary to reach the maximum number of studies. Monographs and dissertations were also considered for review on topics where published studies were scarce. The title, abstract and keywords of the studies in English or Portuguese were read and, if they met the objective of the topic, the study was revised in full. Otherwise, the study was disregarded. This was carried out until each topic was completely written.

## 3. Eggshell Microorganisms: Risks for Poultry Embryos and Chicks

**Figure 3:**
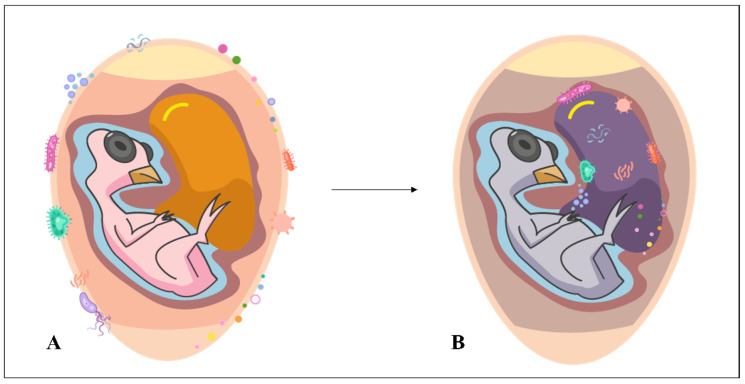
Horizontal contamination in hatching eggs. Microorganisms on the shell (**A**) penetrated and reached the yolk sac (**B**).

Even as an immunologically sensitive embryo, poultry already interact with pathogenic microbes originating from any stage prior to hatching [7]. This interaction may be a consequence of horizontal transmission [8] (Figure 3) and puts the poultry’s life in danger. Fonseca et al. [9] observed that, by contaminating the eggshell, *Campylobacter jejuni* bacteria can penetrate it, cross the albumen and reach the yolk sac, probably resulting in embryonic mortality. This led the authors to state that the immunity conferred by breeding hens to the egg/embryo may be insufficient and inefficient for certain infections. In addition, although the eggshell is an oval antimicrobial wall formed by the fusion of membranes and mineral layers equivalent to a vital organ of a living organism (it promotes the flow of nutrients, water, oxygen and carbon dioxide to keep the embryo alive) [10], it is not totally resistant to microbial entry. The eggshell is challenged when there are microorganisms trying to move from its surface to the main target (embryo). Oliveira et al. [6] reviewed the types of microorganisms that contaminate eggshells. Among the bacterial and fungal genera cited are *Alcaligenes*, *Enterobacter*, *Escherichia*, *Klebsiella*, *Proteus*, *Providencia*, *Pseudomonas*, *Salmonella*, *Clostridium*, *Enterococcus*, *Staphylococcus*, *Streptococcus*, *Aspergillus*, *Candida* and *Penicillium*.

Previously published studies have reported the adverse relationship of microorganisms with embryos and/or chicks. Weil and Volentine [11] reported that contamination of the yolk sac of the chicken embryo by *Shigella dysenteriae* can cause lethal infection. Embryos from chickens killed by contamination with avian pathogenic *Escherichia coli* and *Salmonella enterica subsp. enterica serovar* Enteritidis showed signs of congestion and diffuse redness throughout the skin, head and neck, as well as microscopic lesions in the yolk sac, including congestion, inflammation, damaged blood vessels and abnormal endodermal epithelial cells [1]. Fungi of the genus *Aspergillus*, which may be responsible for causing mycoses or mycotoxicosis, have been isolated from dead chicken embryos [12]. Saleemi et al. [13] reported that aflatoxigenic fungal extracts isolated from *Aspergillus* fungi caused high embryonic mortality, weight reduction and severe alterations in the liver (fatty alteration and cell necrosis) and kidneys (congestion and tubular necrosis) of chicks. Karunarathna et al. [14] demonstrated that multidrug-resistant *Escherichia coli* and *Enterococcus* were recovered from the yolk of non-viable chicken embryos at hatching. Contamination by *Enterococcus* spp. can trigger pulmonary hypertension syndrome in chicken embryos and chicks [15]. Mortality of chicken embryos associated with *Enterococcus* contamination was reported by Karunarathna et al. [16]. Multidrug-resistant bacteria that cause yolk sac infection, including *Escherichia coli*, *Salmonella*, and *Staphylococcus*, have been recovered from dead embryos and chicks [17,18]. Far et al. [19] observed that dead ostrich embryos were contaminated with *Pseudomonas* spp., *Klebsiella* spp., *Bacillus* spp., *Citrobacter* spp., *Staphylococcus* spp., *Proteus* spp., *Aeromonas* spp., *Enterobacter* spp., as well as *Escherichia coli* with antimicrobial resistance profile.

The findings mentioned above raise concerns, especially in relation to the health of poultry and humans, since multiresistant microorganisms can spread and cause massive irreversible damage. In addition, the undue, exacerbated use of sanitizers without proven scientific tests and without the prescription of trained professionals can contribute to even worse health and economic instability. Therefore, collective efforts within the poultry industry should focus on antimicrobial interventions that involve the controlled use of broad-spectrum sanitizers focused on hatching egg sanitization.

## 4. Essential Oils and Their In Vitro Antimicrobial Activity

Essential oils are any aromatic, viscous and volatile oils belonging to plants. *Syzygium aromaticum*, *Allium sativum*, *Ocimum basilicum*, *Thymus vulgaris*, *Lavandula angustifolia*, *Eucalyptus globulus*, *Citrus sinensis*, *Citrus aurantifolia*, *Cinnamomum cassia*, *Rosmarinus officinalis*, *Origanum vulgare*, *Allium cepa*, *Cymbopogon winterianus*, *Cymbopogon flexuosus*, *Piper nigrum*, *Zingiber officinale*, *Protium pallidum*, *Litsea citrata*, *Satureja hortensis*, *Salvia officinalis*, *Mentha piperita*, *Cedrus deodara*, and *Cuminum cymincum* are examples of plant species that provide commercially available essential oils that may have promising futures in poultry nutrition and production such as egg coating additives and sanitizers for hatching eggs. This is because essential oils have a chemical configuration that triggers their biological properties. For example, hydrocarbons, esters, lactones, alcohols, oxides, phenols, ketones, and aldehydes are present in the chemical composition of essential oils with similar or distinct bioactive functions. Depending on the compound, these functions include antimicrobial, antiviral, antitumoral, antibacterial, stimulant, anesthetic, anti-inflammatory, anti-fungal, antipyretic, and spasmolytic [20]. The content, quality, and effectiveness of essential oil compounds depend on factors such as extraction, which can be by hydro distillation, steam distillation, supercritical CO_2_ extraction, ultrasonic extraction, and cold pressing [21,22,23,24].

In vitro antimicrobial screenings initially detect the potential viability of essential oils before they are used as in vivo antimicrobial agents. These screenings demonstrated that essential oils are effective against standard Gram-negative and positive bacterial strains and avian isolates, as well as standard and avian-isolated fungi (Table 1). Among the bacteria are *Salmonella enterica subsp. enterica serovar* Enteritidis, *Salmonella enterica subsp. enterica serovar* Typhimurium, *Salmonella enterica subsp. enterica serovar* Infantis and avian pathogenic *Escherichia coli* (APEC), which are important pathogenic bacteria for poultry and public health (Table 1). The antimicrobial effectiveness of essential oils ranges from mild to very strong. In fact, some of them have been shown to be more effective than conventional antibiotics [25,26]. Thymol, eugenol, carvacrol, linalool, citral, limonene, trans-cinnamaldehyde, geraniol and citronellal are some compounds that are part of the composition of some essential oils that can act as protagonists in antimicrobial action (Table 1). The main mechanisms responsible for making the bacterial [27] and fungal [28] cells unfeasible are listed below:

Bacteria:

1. Cell membrane alteration and increased permeability.
2. Stops energy production.
3. Blocks active transport.

Fungi:

1. Cell membrane disruption, alteration, and inhibition of cell wall formation.
2. Dysfunction of fungal mitochondria.
3. Inhibition of efflux pumps.

Essential oils can promote beneficial actions for human health by reducing pain and inflammation, protecting and healing wounds, neutralizing or stopping the development of carcinogens, neutralizing oxidative stress and possessing antiviral, antibacterial, antifungal, cardioprotective, antidiabetic, and insect-repellent properties; among other benefits, they can also potentially treat central-nervous-system-based disorders [29,30,31,32]. The safety of a stock of essential oils including *Ocimum basilicum*, *Zingiber officinale*, *Lavandula officinalis*, *Cymbopogon citratus*, *Mentha piperita*, *Rosmarinus officinalis*, *Thymus vulgaris*, *Eugenia caryophyllata*, and *Allium sativum* has been documented and they received the generally recognized as safe (GRAS) seal [33]. However, the intake of essential oils needs to be monitored, as they can, like any other edible food, cause an inappropriate effect.

**Table 1:** Microorganisms sensitive to essential oils through in vitro screening.

Essential Oil	Majority Element	Analysis	Method	Microorganism	Origin Microorganism	Study
*Thymus vulgaris* *Origanum vulgare* *Mentha pulegium*	-	B*	Disk diffusion	*Bacillus cereus* *Clostridium perfringens* *Enterococcus faecalis* *Enterococcus faecium* *Escherichia coli* *Listeria monocytogenes* *Pseudomonas aeruginosa* *Salmonella enterica subsp. enterica serovar* Enteritidis *Staphylococcus aureus* *Staphylococcus epidermidis*	ATCC	[34]
*Allium sativum*	Diallyl disulfide (44.6%)	B		*Salmonella enterica subsp. enterica serovar* Typhimurium *Yersinia enterocolitica* *Bacillus cereus* *Staphylococcus aureus*	ATCC and NCTC	[35]
*Cinnamomum cassia* *Syzygium aromaticum*	Eugenol (72.13%) Eugenol (83.63%)	B	Agar dilution	*Escherichia coli* *Staphylococcus aureus* *Pseudomonas aeruginosa*	ATCC and human clinical isolate	[36]
*Thymus vulgaris* *Foeniculum vulgare* *Cuminum cyminum*	-	B and F*	Disk diffusion	*Escherichia coli* *Staphylococcus aureus* *Pasteurella multocida* *Salmonella enterica subsp. enterica serovar* Typhimurium *Aspergillus fumigatus* *Candida albicans*	MTCC	[37]
*Origanum vulgare* *Origanum majorana*	-	B	Disk diffusion and broth microdilution	*Staphylococcus aureus*	Poultry meat	[38]
*Thymus vulgaris* *Origanum vulgare*	Thymol (41.60%) Carvacrol (53.4%)	B	Broth microdilution	*Bacillus cereus* *Staphylococcus aureus* *Salmonella enterica subsp. enterica serovar* Infantis *Escherichia coli*	Clinical isolate and poultry meat isolate	[39]
*Lippia rotundifolia* *Lippia origanoides*	-	B	Disk diffusion and dilution	*Staphylococcus aureus* *Escherichia coli*	Poultry feces	[40]
*Thymus schimperi* *Rosmarinus officinalis* *Eucalyptus globulus*	Carvacrol (71.02%) α-Pinene (50.83%) 1,8-Cineole (63.00%)	B	Well diffusion	*Streptococcus pyogenes* *Staphylococcus epidermidis* *Salmonella enterica subsp. enterica serovar* Typhimurium *Shigella* spp. *Pseudomonas aeruginosa* *Staphylococcus aureus* *Escherichia coli* *Trichophyton* spp. *Aspergillus* spp.	-	[41]
*Pimenta pseudocaryophyllus* Citrus Terpenes	- Limonene (28.67%)	B	Disk diffusion	*Salmonella enterica subsp. enterica serovar* Enteritidis *Escherichia coli* *Staphylococcus aureus* *Listeria innocua* *Enterococcus faecalis*	ATCC	[42]
*Lavandula* × *intermedia* *Lavandula angustifolia*	Linalool (57.10%) Linalool (53.97%)	B and F	Well diffusion and broth microdilution	*Bacillus cereus* *Bacillus pumilus* *Enterococcus faecalis* *Escherichia coli* *Klebsiella oxytoca* *Klebsiella pneumoniae* *Kocuria rhizophila* *Listeria monocytogenes* *Proteus mirabilis* *Pseudomonas aeruginosa* *Salmonella enterica subsp. enterica serovar* Enteritidis *Staphylococcus aureus* *Streptococcus pyogenes* *Yersinia enterocolitica* *Candida albicans* *Candida glabrata* *Candida kefyr* *Candida krusei* *Candida tropicalis* *Cryptococcus neoformans* *Hansenula anomala* *Saprochaete capitate* *Microsporum canis* *Microsporum gypseum* *Trichophyton mentagrophytes* *Trichophyton rubrum* *Aspergillus fumigatus* *Aspergillus niger* *Fusarium oxysporum* *Penicillium citrinum*	ATCC, NCTC, and food and clinical isolates	[43]
*Kaempferia galanga* *Cymbopogon flexuosus* *Pogostemon cablin* *Curcuma caesia* *Cymbopogon winterianus* *Clausena heptaphylla* *Cinnamomum tamala* *Ocimum sanctum* *Cinnamomum camphora*	P-Methoxycinnamate (27.84%) Geranial (Citral a) (42.14%) Patchouli alcohol (32.33%) Eucalyptol (15.05%) Citronellal (38.68%) (E)-Anethole (53.49%) Eugenol (72.33%) Eugenol (41.89%) Camphor (49.43%)	B and F	Disk diffusion and broth dilution	*Staphylococcus aureus* *Bacillus cereus* *Bacillus subtilis* *Salmonella enterica subsp. enterica serovar* Typhimurium *Escherichia coli* *Aspergillus niger* *Aspergillus fumigatus* *Saccharomyces cerevisiae Candida albicans*	ATCC	[44]
*Aloysia triphylla* *Cinnamomum zeylanicum* *Cymbopogon citratus* *Litsea cubeba* *Mentha piperita*	Limonene (*E*)-Cinamaldeído Neral Geranial Mentol	B and F	Disk diffusion and broth microdilution	*Salmonella enterica subsp. enterica serovar* Enteritidis *Salmonella enterica subsp. enterica serovar* Typhimurium *Saccharomyces cerevisiae*	Poultry	[45]
*Syzygium aromaticum*	-	B	Disk diffusion and dilution	Avian pathogenic *Escherichia coli* *Escherichia coli* *Salmonella enterica subsp. enterica serovar* Enteritidis *Salmonella* spp.	Poultry	[46]
*Satureja kitaibelii*	p-Cymene (24.4%)	B	Broth microdilution	*Escherichia coli* *Staphylococcus aureus*	ATCC	[47]
*Origanum vulgare*	Germacrene D (21.5%)					
*Achillea millefolium*	Camphor (9.8%)					
*Achillea clypeolata*	1,8-Cineole (45.1%)					
*Thymus serpyllum*	Geraniol (63.4%)					
*Origanum vulgare*	Carvacrol (66.98%)	B	Broth microdilution	*Salmonella enterica subsp. enterica* serovar Infantis	Intensive poultry farms (boot swabs)	[48]
*Melaleuca alternifolia*	Terpinen-4-ol (>30%)	B and F	Modified zone of inhibition test with glass cylinders	*Mycobacterium smegmatis* *Staphylococcus epidermidis* *Staphylococcus aureus* *Methicillin-resistant Staphylococcus aureus* *Streptococcus pyogenes* *Pseudomonas aeruginosa* *Antibiotic-resistant Pseudomonas aeruginosa* *Bordetella bronchiseptica* *Klebsiella pneumoniae* *Candida albicans*	ATCC	[49]
*Rosmarinus officinalis*	1,8-Cineole (>30%)					
*Cinnamomum cassia*	Trans-cinnamaldehyde (>30%)					
*Cymbopogon flexuosus*	Citral (81.84%)	B and F	Disk diffusion and serial dilution	*Escherichia coli* *Escherichia coli* *Salmonella enterica subsp. enterica serovar* Typhimurium *Proteus vulgaris* *Pseudomonas aeruginosa* *Staphylococcus aureus* *Streptococcus faecalis* *Bacillus subtilis* *Xanthomonas oryzae* *Xanthomonas malvacearum* *Aspergillus niger* *Fusarium oxysporum* *Fusarium udum* *Magnaporthe grisea*	NCIM and isolated from blight and blast infected leaves	[50]
*Cymbopogon martini*	Geraniol (63.79%)					
*Eucalyptus citridora*	Citronellal (76.80%)					
*Pelargonium* spp.	Geraniol (22.38%)					
*Cymbopogon winterianus*	Citronellal (34.10%)					
*Satureja hortensis*	Thymol (41.28%)	B	Disk diffusion and broth microdilution	*Escherichia coli* *Salmonella enterica subsp. enterica serovar* Enteritidis	Poultry infections	[51]
*Origanum vulgare*	Carvacrol (65.80%)	B and F	Disk diffusion	*Staphylococcus aureus* *Escherichia coli* *Candida albicans*	ATCC	[52]
*Melaleuca alternifolia*	Terpinen-4-ol (39.60%)					
*Citrus limonum, Cinnamomum cassia, Eugenia caryophyllus, Eucalyptus globulus, and Rosmarinus officinalis*	-					
*Ocimum basilicum*	Linalool (65.20%)					
*Crithmum maritimum*	γ-Terpinene (32.9%)	B and F	Well Diffusion and broth microdilution	*Escherichia coli* *Listeria *monocytogenes** *Staphylococcus aureus* *Candida *albicans** *Pseudomonas fluorescens*	ATCC and DSMZ	[53]
*Cuminum cyminum*	Cumin aldehyde (30.2%)					
*Cupressus arizonica*	α-Pinene (41.0%)					
*Pimpinella anisum*	(E)-Anethole (96.7%)					
*Zingiber officinale*	-	B	Disk diffusion	*Escherichia coli* *Staphylococcus aureus*	ATCC	[54]
*Citrus aurantifolia*						
*Cymbopogon citratus*						
*Origanum vulgare*	Carvacrol (68.72%)	B	Broth microdilution	*Staphylococcus aureus* *Listeria monocytogenes* *Escherichia coli* *Salmonella enterica subsp. enterica serovar* Typhimurium *Staphylococcus aureus* *Listeria monocytogenes* *Escherichia coli* *Salmonella* spp.	ATCC and isolated food	[55]
*Thymus vulgaris*	Thymol (54.60%)					
*Eugenia caryophyllus*	Eugenol (86.25%)					
*Cinnamomum cassia*	Trans-cinnamaldehyde (86.57%)					
*Ocimum basilicum*	Estragole (60.98%)	B	Broth microdilution	*Escherichia coli* *Staphylococcus aureus*	ATCC	[56]

*B, Antibacterial; *F, Antifungal; ATCC, American Type of Culture Collection; MTCC, Microbial Type Culture Collection; NCTC, National Collection of Type Cultures; NCIM, National Collection of Industrial Microorganisms; DSMZ, German Collection of Microorganisms and Cell Culture. Only essential oils that inhibited the growth of all bacteria/fungi in each study were cited in the table. When the study used two antimicrobial screening methods, we considered that the oil was efficient when it inhibited microorganisms in at least one of them. The information was collected on 26 April 2023.

## 5. Antimicrobial Effect of Essential Oils In Vivo (Eggshells)

Sanitization is the basis that sustains microbial control in hatching eggs. By prioritizing sanitization, the poultry industry prevents contamination between hatching eggs themselves and between hatching eggs, humans and poultry. This minimizes or nullifies the risk of pathogenic contamination to poultry and human lives. Thus, sanitizers that combine at least bactericidal or bacteriostatic and fungicidal or fungistatic characteristics are compatible options for sanitizing hatching eggs. As shown in Table 1, microorganisms that can colonize the eggshells [6] showed to be sensitive to the action of different essential oils. This is supported by in vivo tests, which have shown that essential oils reduce the total count of mesophilic aerobic bacteria, enterobacteria, molds and yeasts (Table 2). In addition, essential oil components such as carvacrol, eugenol and trans-cinnamaldehyde at 0.25%, 0.5% and 0.75% when applied to eggs by immersion showed the potential to inactivate *Salmonella enterica subsp. enterica serovar* Enteritidis in eggshells with or without organic matter [57]. More importantly, the ability of trans-cinnamaldehyde to block the migration of this microorganism into the egg contents has been suggested [58]. This evidence may support observed or suggested findings that chicken embryos from eggs sanitized with essential oils or their compounds have a reduced microbial load [59,60].

Essential oils also contribute to the self-sanitization of eggshell surfaces when used as a bioactive element in coatings applied by spraying or immersion and permanently formed on the surface of eggs [54,67,68]. This was associated with a microbial reduction of eggshells and egg contents [56,67]. Its antimicrobial effect seems to remain active for longer periods [5,54,59,67], protecting the eggs from microbial recontamination, without the need for additional applications. Frequently reapplication of a sanitizer to control egg contamination is not ideal for two reasons: the cost and because sanitizing during incubation can have undesirable effects on the embryos and reduce hatchability as reported in hatching eggs sprayed with albumin at different incubation periods [69]. In addition to the fact that, in the early stages of incubation, embryos are particularly sensitive to sanitizers such as formaldehyde [70], it is hypothesized that the application of sanitizers during incubation may influence eggshell temperature, particularly if applied by liquid, which interferes with the proper development of the embryo. Therefore, it is recommended to use sanitizers that do not require continuous reapplication, such as those based on essential oils.

## 6. Toxicity of Essential Oils for Poultry Embryos and Chicks

Before essential oils are effectively used for a specific purpose within the poultry chain, it is advisable to consult scientific studies that prove the limits of the safe use of essential oils. Although essential oils have beneficial antimicrobial residual effects on hatching eggs [5,71], care must be taken to ensure that their contact with embryos and chicks does not cause permanent damage that limits their behavior, physiology, morphology and, above all, their survival. Embryo development with successful hatching is the first positive sign of evaluating a sanitizer. In advance, essential oils should have a positive evaluation, as it has been reported that hatchability rates of hatching eggs sanitized with essential oils can be improved by up to 12.59% [5]. However, so that this preliminary assessment can be better supported, we review the toxic or non-toxic effects of essential oils on embryos and chicks below.

According to de Oliveira [72], spraying of *Melaleuca alternifolia* essential oil at 0.75% on the shells or its delivery in the air chamber of hatching eggs did not affect the viability, heart rate, probability of occurrence of malformation, or the developmental stage of chicken embryos. However, the probability of survival was significantly reduced when this oil was injected into the air chamber. Morphological abnormalities in embryos/chicks from eggs sprayed with essential oils have been reported [72,73], but according to de Oliveira [72], they were within normal limits. Demirci et al. [74] reported that the application of *Origanum onite* essential oil at 250 µg/pellet strongly irritated the chorioallantoic membrane. They stated that this was due to thymol (11.6%) present in the oil composition. Essential oil compounds can negatively affect poultry embryos depending on how they are applied. A dose of 50 µM Citral caused embryonic malformation [75] and a dose of carvacrol (50 μg/kg) impaired the normal development of embryos [76] when injected in ovo. These effects are induced based on concentration [75,76]. On the other hand, Ulucay and Yildirim [77] suggested that embryo respiration and quail chick weight were not affected after egg sanitization with 1% thymol, carvacrol, or cinnamaldehyde. Thus, the chemical composition and route of application of essential oils are factors that can have a significant influence on embryo safety.

**Figure 4:**
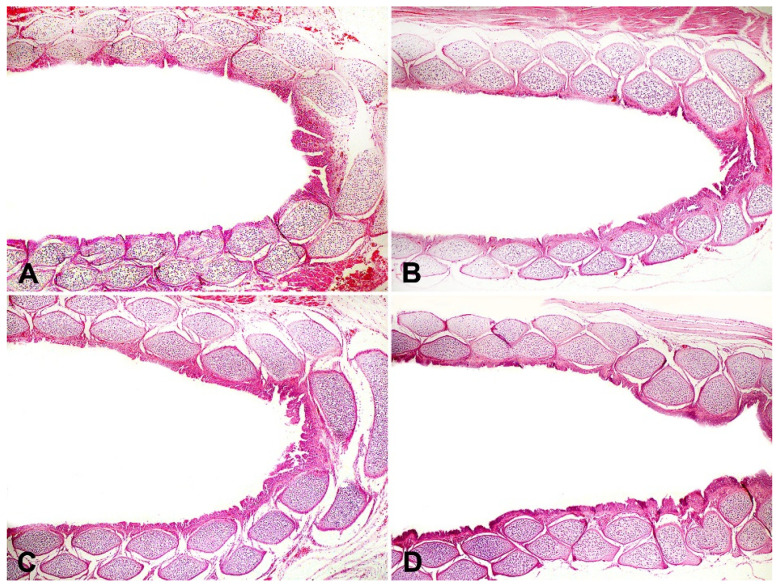
One-day-old chicks. Tracheas showing no histological changes (H&E objective 4×). (**A**) Chick from non-sanitized eggs. (**B**) Chick from eggs sanitized with *Syzygium aromaticum* essential oil. (**C**) Chick from eggs sanitized with grain alcohol. (**D**) Chick from eggs sanitized with paraformaldehyde. No significant difference among treatments tested.

**Figure 5:**
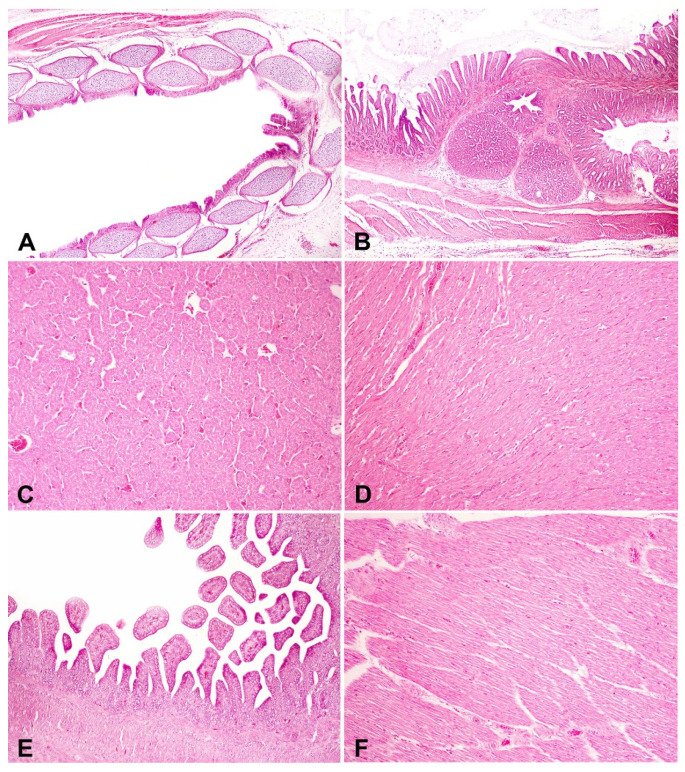
One-day-old chicks. Organs showing no histological changes. (**A**) Trachea, chick from eggs sanitized with paraformaldehyde (H&E objective 4×). (**B**) Proventriculus and gizzard, chick from non-sanitized eggs (H&E objective 4×). (**C**) Liver and gallbladder, chick from eggs sanitized with grain alcohol (H&E objective 10×). (**D**) Heart, chick from eggs sanitized with paraformaldehyde (H&E objective 10×). (**E**) Intestine, chick from eggs sanitized with *Syzygium aromaticum* essential oil (H&E objective 10×). (**F**) Chest, skeletal muscle, chick from eggs sanitized with grain alcohol (H&E objective 10×). No significant difference among treatments tested.

*Syzygium aromaticum* essential oil at 0.39%, when applied to hatching eggs, did not cause alterations or lesions in the trachea of day-old chicks (Figure 4) showing that the application of this compound to hatching eggshells in pre-incubation and without re-application during incubation had a protective effect and probably did not cause any tracheal tissue disturbance that compromised the respiratory system of day-old chicks. Furthermore, in the histological analysis of tissues and organs (large and small intestines, pectoral muscle, proventriculus and gizzard, liver and gallbladder, and heart), no microscopical changes were detected (Figure 5). The lack of morphological changes in the tissue samples supports the absence or negligible topical toxicity of *Syzygium aromaticum* essential oil in ensuring the hatching of healthy chicks.

Other published studies have reinforced that most essential oils do not have negative effects on embryos and chicks, even when injected directly into the developing embryo (Table 3).

## 7. Comparing Essential Oils and Formaldehyde for Sanitizing Hatching Eggs

Formaldehyde is still preferably used in the practice of sanitizing hatching eggs [3,85,86]. Antimicrobial effectiveness and cost are two of the main reasons why formaldehyde remains in use in the poultry industry. Even its strong toxicity to poultry embryos [66,85,87] and humans [88,89,90] has not managed to have it removed from the practice of sanitizing hatching eggs. However, researchers are strongly committed to continuing to alert the poultry industry that, from a health point of view, formaldehyde is not compatible with a sustainable and safe poultry chain.

The sanitization of hatching eggs with natural sanitizers is based on a sanitary practice of microbial control of eggshells without synthetic chemical treatments, which aims to contribute to the production of healthy chicks free of pathogenic microorganisms using exclusively substances derived from plants and friends of living organisms [5,64,91,92]. Comparing natural sanitizers made from essential oils with synthetic sanitizers made from formaldehyde, there should be conscious support for the transition from sanitization systems that involve aggressive products to those that use green and responsible products. In addition to the antibiotic profile capable of significantly reducing the microbial count of hatching eggshells, one of the main advantages of using essential oils as sanitizers for hatching eggs is the productive results promoted in terms of hatchability, which, on average, are not inferior to those of sanitization with formaldehyde (Table 4). Thus, the application of essential oils to hatching eggs does not require additional or different practices to promote the production of the same number of poultry than is routine in the conventional poultry sector. The prioritized use of synthetic chemicals in hatching egg management can be minimized by replacing them with essential oils.

## 8. Conclusions

In general, we have found that essential oil sanitizers are effective in reducing the microbial load on eggshells. From a safety point of view, the direct application of essential oils in developing poultry can generate toxic effects on the survival and integrity of these animals, but this seems to be mainly associated with specific components of the composition of essential oils and/or factors intrinsic to the application protocols, such as method, time, location and concentration. This raises the hypothesis that the residual contact of essential oils applied on hatching eggshells with the embryo is minimal and gradual, as most of the effects found when these compounds were applied to eggshells were beneficial for the embryo and chick. The dosage and concentration of the essential oils in contact with the embryos need to be studied and adjusted, especially if applied directly so that all harms are converted into benefits. The effectiveness of essential oils is comparable to formaldehyde, but they are less toxic. Complementary studies on the microbiological profile of embryos and chicks hatched from eggs sanitized with essential oils need to be carried out. In addition, the economic viability of essential oils before their possible effective use in the sanitization of hatching eggs needs to be investigated to know which essential oils adapt to small- and large-scale applications.

## Figures and Tables

**Table 2:** Eggshell microbial counts reduced by actions of essential oils.

Essential Oil or Its Component	Essential Oil Concentration	Eggshell Application	Egg Type	Eggshell Contamination	Eggshell Microbial Load	Study
Carvacrol	0.25, 0.5 and 0.75%	Immersing	Chick	Inoculation	*Salmonella enterica subsp. enterica serovar* Enteritidis	[57]
Eugenol						
Trans-cinnamaldehyde						
*Thymus vulgaris*	0.25, 0.5 and 1 mg/mL	Immersing	Chick	Inoculation	*Salmonella enterica subsp. enterica serovar* Enteritidis CICC 21482 *Salmonella enterica subsp. enterica serovar* Typhimurium CICC 22956	[61]
*Cymbopogon winterianus*	0.1, 0.15 and 0.2%	Spraying	Chick	Natural	Molds and yeasts	[62]
*Cymbopogon flexuosus*	1%	Immersing	Chick	Natural	Total coliforms Yeast and filamentous fungi Aerobic mesophylls	[63]
*Lippia rotundifolia*						
*Syzygium aromaticum*	0.39%	Spraying	Chick	Natural	Total aerobic mesophilic bacteria Enterobacteriaceae	[64]
*Syzygium aromaticum*	10–80 µg/g	Vaporizing	Chick	Inoculation	*Escherichia coli* *Salmonella enterica subsp. enterica serovar* Typhimurium *Staphylococcus aureus*	[65]
				Natural	Bacterial counts	
*Origanum vulgare*	0.5%	Immersing	Chick	Natural	Total bacterial	[66]
*Cuminum cyminum*						
Trans-cinnamaldehyde	0.48%	Immersing	Chick	Inoculation	*Salmonella enterica subsp. enterica serovar* Enteritidis	[58]

**Table 3:** Effects of direct or non-direct application of essential oils on embryos or chicks.

Essential Oil or Its Component	Essential Oil Concentration	Egg Application Method	Application Target	Egg Type	Authors’ Findings for Embryos and Chicks	Study
*Origanum* *vulgare*	0.2 and 0.4% or 0.5%	Sanitizing	Eggshell	Chick	Improved hatch time and chick body weight. Heavy and well-developed embryos. No brain and spinal cord malformations in the embryos.	[66,71,78]
*Cuminum cyminum*						
*Juniperus* *excelsa*	10% ratio of 9 (oil):1 (ethyl alcohol)	Micropipetting	Blastodisc	Chick	Antiangiogenic action.	[79]
*Cymbopogon winterianus*	0.1, 0.15 and 0.2%	Sanitizing	Eggshell	Chick	No influence on chick quality and weight.	[62]
*Syzygium* *aromaticum*	0.39%	Sanitizing	Eggshell	Chick	No weight changes and atrophy or hypertrophy in large and small intestine, pectoral muscle, proventriculus and gizzard, liver and gallbladder and heart of embryos and day-old chicks.	[60]
*Origanum* *vulgare*	0.5%	Sanitizing	Embryo	Chick	No embryonic malformations. Restored the antioxidant balance of the embryos.	[80]
Commercial blend	0.2 mL ratio of 2 (saline): 1 (Commercial blend)	Injecting	Amnion	Chick	Chick length reduction. Improvement of intestinal morphometric properties of broiler chickens. No adverse effect on growth performance.	[81]
*Thymus* *vulgaris*	0.03 mL/egg	Injecting	Embryo	Chick	Improved the initial weight of chicks.	[82]
*Rosmarinus* *officinalis*	1 µL or 3 µL/egg	Injecting	Air chamber	Quail	Embryo protection (better embryonic development) and higher birth weight.	[83]
Trans-cinnamaldehyde	0.48%	Washing	Eggshell	Chick	No effect on yolk sac, embryo and tibia weight. No change in embryo and tibia length.	[84]

**Table 4:** Comparison between the efficiency of essential oils and formaldehyde after application in hatching eggs.

Compounds	Bacterial Count (log) ^a^	Hatchability (%) ^a^	Significance ^b^	Most Efficient	Study
*Origanum onites*	<0.47	>1.98	* TBC ^ns^ Hatchability	Essential oil	[93]
Formaldehyde	<0.06	>1.89			
*Thymus vulgaris*	<1.68	>6.95	*	Formaldehyde	[94]
Formaldehyde	<1.81	>9.70			
*Syzygium aromaticum*	<1.19	>10.66	ns	Similar	[64]
Paraformaldehyde	<1.26	>7.84			
*Origanum vulgare*	<6.33	>12.05	*	Essential oils	[66]
*Cuminum cyminum*	<6.13	>11.70			
Formaldehyde	<3.03	<2.01			

^a^ Comparison of essential oils and formaldehyde with non-sanitized eggs; ^b^ Comparison between essential oil and formaldehyde; * Significant; ^ns^ non-significant, TBC, Total bacteria count.

## Data Availability

Not applicable.
